# Referral patterns surrounding the COVID-19 pandemic to a child and adolescent mental health clinic in Gauteng, South Africa

**DOI:** 10.3389/fpsyt.2026.1894740

**Published:** 2026-08-24

**Authors:** Justin Kuni, Lihlithemba Sigudhla, Reitumetse Chaisi, Puleng Raboroko, Izabella Carvalho, Malibongwe Madi, Hazel Madonsela, Jennifer Stead, Ronelle Price-Hughes, Yvette Nel

**Affiliations:** 1 The unit for undergraduate medical education (UUME), University of the Witwatersrand, Johannesburg, South Africa; 2 Department of Psychiatry, University of Witwatersrand, Johannesburg, South Africa

**Keywords:** child and adolescent psychiatry, COVID-19, mental health, out-patient clinic, referral patterns

## Abstract

The COVID-19 pandemic and related lockdown period had global health and psychosocial consequences, with specific effects on the mental health and development of children and adolescents. However, little is known about the impact of this period on longitudinal patterns of presentation to mental health services in children and adolescents. The aim of this study is to describe the patterns of referrals to a single tertiary specialist child and adolescent mental health clinic in Johannesburg, Gauteng, South Africa, before, during, and after the national COVID-19 lockdown. Data comprised a longitudinal analysis of 739 complete telephonic referral forms for children and adolescents, ages 4 to 18, over a four-year period, from 1 April 2019 to 31 March 2023. There was an initial inverse relationship between lockdown stringency and referral numbers. As restrictions eased, a gradual increase in referral activity was observed. This pattern did not continue with subsequent lockdown level adjustments later in 2020 and onwards. There were more male referrals (62%) across all periods compared to females (38%), with a noted rise in female referrals during, and after lockdown. Mood-related symptoms increased from 21.97% pre-lockdown to 38.36% during lockdown year 1 (p = 0.0022). Anxiety-related symptoms also increased significantly (p = 0.0007). There was an initial absence of substance related symptoms during total restrictions of movement and ban on alcohol, with a subsequent increase in year two of lockdown and post-lockdown. Disruptive, impulse control and conduct symptoms decreased during lockdown year 1 (p = 0.0004), as did neuro-developmental related symptoms (p=0.0005). These findings suggest a possible pandemic-related shift in symptoms and referral patterns in a tertiary child and adolescent out-patient setting and are explored within the context of health seeking behavior and pathways to care during the COVID-19 pandemic in South Africa. Relevant socioeconomic changes impacting child and adolescent mental health and access to care during this period are described as plausible contributing factors to these patterns. This analysis, while limited and not generalizable, contributes to the existing literature reflecting patterns during the COVID-19 pandemic and related lockdowns in child and adolescent mental health, providing data for future planning and support.

## Introduction

The COVID-19 pandemic, and related lockdowns, created major global challenges for economies and healthcare systems, including mental health system disruption (1). Declared a pandemic by the WHO in March 2020, the outbreak of COVID-19 led to worldwide lockdowns. The pandemic and response with regard to lockdowns in South Africa largely followed international trends (2, 3). While deemed necessary to curb infection, these lockdowns had significant psychosocial effects, particularly on children and adolescents, with many experiencing fear, isolation, and disrupted routines (4–6). There is existing evidence to suggest significant mental health effects in children and adolescents, largely due to the psychosocial impact of the COVID-19 pandemic and related lockdowns (1, 4–6). Apart from the psychosocial impact of the pandemic, there are also proposed biological mechanisms suggested as to how the SARS CoV2 virus may directly impact on neurological and neuropsychiatric symptoms (7). Although it is widely agreed that children are mostly asymptomatic when infected with COVID-19, or present with mild disease with low mortality rates, children may present with post-infectious inflammatory syndromes (8). Reports of post-COVID neuropsychiatric symptoms in children suggest a multifactorial etiology, with evidence of direct neural pathways combined with the impact of profound psychosocial disruption (9).

In South Africa, COVID-19 and related lockdowns resulted in severe social, economic and political disruptions, with marked decrease in utilization and access to health care, due to factors such as fear of contracting COVID-19, limited transportation to and from healthcare facilities, overburdened health care facilities, and closure of non-essential services, in an already strained health care system with historical inequality and disparities in health care access (1, 3, 10, 11). The pandemic also specifically affected access to mental healthcare. National drug shortages and disruptions in the supply chain of medications used to treat mental illnesses exacerbated relapses (10). Measures to mitigate the effects of the lockdown were implemented, such as continuing to offer all mental health services with COVID-19 preventative measures in place, but this was not sustainable and thus ultimately, access to mental health services was disrupted (10).

Subramaney et al. outlined the risks associated with the pandemic on child and adolescent mental health early in the pandemic (12). The social and economic impact of the COVID-19 pandemic and related lockdowns was noted to disproportionally affect children and adolescents in poorer communities globally, who were unable to attend school altogether due to decreased access to virtual schooling (12, 13). Many South African children depend on school feeding programs and rates of malnutrition among children increased during the lockdown because school feeding programs were suspended along with the closure of schools (12). The national lockdown disproportionally impacted children and adolescents already living in poverty and increased the number of children and adolescents living in poverty due to sudden unemployment. Poverty and unemployment exacerbate domestic violence and gender-based violence. Since movement was restricted during the lockdown, these children and adolescents had no place to go, such as school or aftercare programs, to escape abuse (14). Children were also at higher risk for sexual violence, and the services that could prevent or support the victims were halted, exacerbating the mental health consequences of abuse (14). Lockdowns also increased uncontrolled screen time and social media usage, providing a novel threat to mental health in children and adolescence (15). In line with the increased psychosocial risk, previous reports suggest increased rates of depression and anxiety, as well as behavioral symptoms in children following the implementation of lockdowns (14–17) Internationally, however, some children also reported improved well-being during lockdowns due to reduced academic stress and greater parental support (18). In the early stages of the pandemic in South Africa, Bloom et al. explored the changes that parents noted in their children and adolescents while highlighting the relevant contextual stressors in this population, with parents reporting that children experienced higher mental health problems than adolescents, specifically emotional dysregulation, behavioral problems, and hyperactivity. However, both adolescents and children had evident mental health problems (19).

Mental health referrals may be used to track changes in patterns of mental health symptoms and numbers. Patterns of referrals are difficult to interpret across different international settings due to differences in health care systems, referral pathways and collection of data, however, globally, mental health referrals for adolescents dropped sharply during the initial lockdown in March 2020, as seen in Ireland, where all child and adolescent mental health referrals fell by 11%, with similar reports in other high-income countries and a dearth of literature from low and middle income countries (LMIC) (20, 21). This decline was likely due to restrictions on personal movement, fear of infection, reduced healthcare access, and workforce shortages (22). Stewart et al. noted similar trends in initial decreases in all referrals for child and adolescent mental health services, with disproportionately larger decreases in referrals from disadvantaged communities in a high-income setting (21). As the pandemic progressed, there was an increase in the presentation of children and adolescents to healthcare facilities. This was due in part to relaxed restrictions to allow some movement, but also suggested that the prolonged lockdown conditions may have exacerbated mental health challenges in children and adolescents (20). There were also documented changes in symptom patterns, gender patterns and referral numbers throughout the pandemic in children and adolescents internationally (18). There were several international reports of proportionally increased referrals for females, with increased referrals for anxiety, depression and emotional problems, and decreased referrals for externalizing symptoms such as ADHD and conduct disorder (23–25). A South African regional community organization in the Western Cape province documented similar patterns pre-pandemic and during the pandemic with regards to referrals seeking support for emotional and behavioral challenges and outlined their unique program which increased provision of support during the pandemic for these children and adolescents (26). This service was limited to a small geographic area. There is no published data on the longitudinal trends in child and adolescent mental health care referrals in the context of the COVID-19 pandemic and lockdown in South Africa, and other LMIC.

South Africa has a split public and private health care system, with disproportionate access to health care based on access to funding or insurance for private health care, further increasing historical inequality. The country also faces critical shortages of mental health practitioners and poor overall access to quality mental health services (27). This is especially significant in the child and adolescent mental health care services, where there are few trained subspecialists, and disproportionate access to these subspecialty services in the public and private sectors. Ideally, the first point of contact for patients should be at level 1 (informal and formal primary health services) and then up referral to level 2 or 3 depending on the complexity of the problem, the type of assessment, and/or the type of intervention needed, and then down referred again when stabilized (28). In Southern Gauteng, the movement between the different tiers has not been formally implemented and is not enforced. Patients can therefore access services at any level within their catchment areas. Public sector specialized child and adolescent mental health services are predominantly offered through hospitals even though South Africa’s mental health policy supports community-based care, as there are no child and adolescent psychiatrists outside of hospital settings in the public health care system in Southern Gauteng (29). Within Southern Gauteng there are four hospital based dedicated child and adolescent psychiatry outpatient units within the public sector, with several district mental health clinics providing mental health services to children and adolescents, where patients are seen by registrars training in psychiatry with senior telephonic support.

Longitudinal analysis of referral patterns in child and adolescent settings may provide guidance on trends and changes in mental health care symptoms in this vulnerable population, although cannot provide causal associations with COVID infection, lockdowns, and the psychosocial impact of the pandemic response. Reviewing available data may help with strategic preparation for future pandemics by drawing insights from historical experiences. The aim of this study was therefore to describe the patterns of referrals to a specialist child and adolescent mental health care clinic before, during and after the national COVID-19 lockdown period.

## Materials and methods

### Study design and setting

This study comprised a retrospective longitudinal quantitative descriptive analysis of referral intake screening assessments at a child and adolescent mental health clinic and was conducted at a single tertiary unit, Tara Hospital, in the child and adolescent outpatient department. Tara Hospital is a specialized tertiary government psychiatric facility, which offers adult, child and adolescent, in- and out-patient services to the central Gauteng area, and nationally where specialist services may be lacking.

### Data source

In this study, data were retrieved from the structured telephonic referral form, which is used in routine clinical practice to screen children and adolescents in need of psychiatric care. To be seen at the Tara Hospital Child and Adolescent outpatient unit, caregivers need to contact the clinic during office hours and request to speak to a nursing sister who completes a brief telephonic referral form to determine if the client resides in the correct catchment area and if it is an appropriate referral. Clients from outside the catchment area are directed to the appropriate clinic or unit, in case of an emergency they are directed to their nearest emergency room or casualty and if the main concern is rather educational the caregivers will be directed to an educational psychologist.

The brief telephonic referral form includes demographics, date of birth, school details, language, contact details for caregivers, source of referral, reason for referral, as well as previous assessments by mental health practitioners. Following this screening, if the patient falls into the correct catchment area and requires psychiatric interventions, forms will be given to the caregiver for the schoolteachers to complete. After receiving the complete forms, the patients and caregivers will be given a file number, and booked for a detailed interview which lasts about 3–5 hours.

### Study population

**Figure 1:**
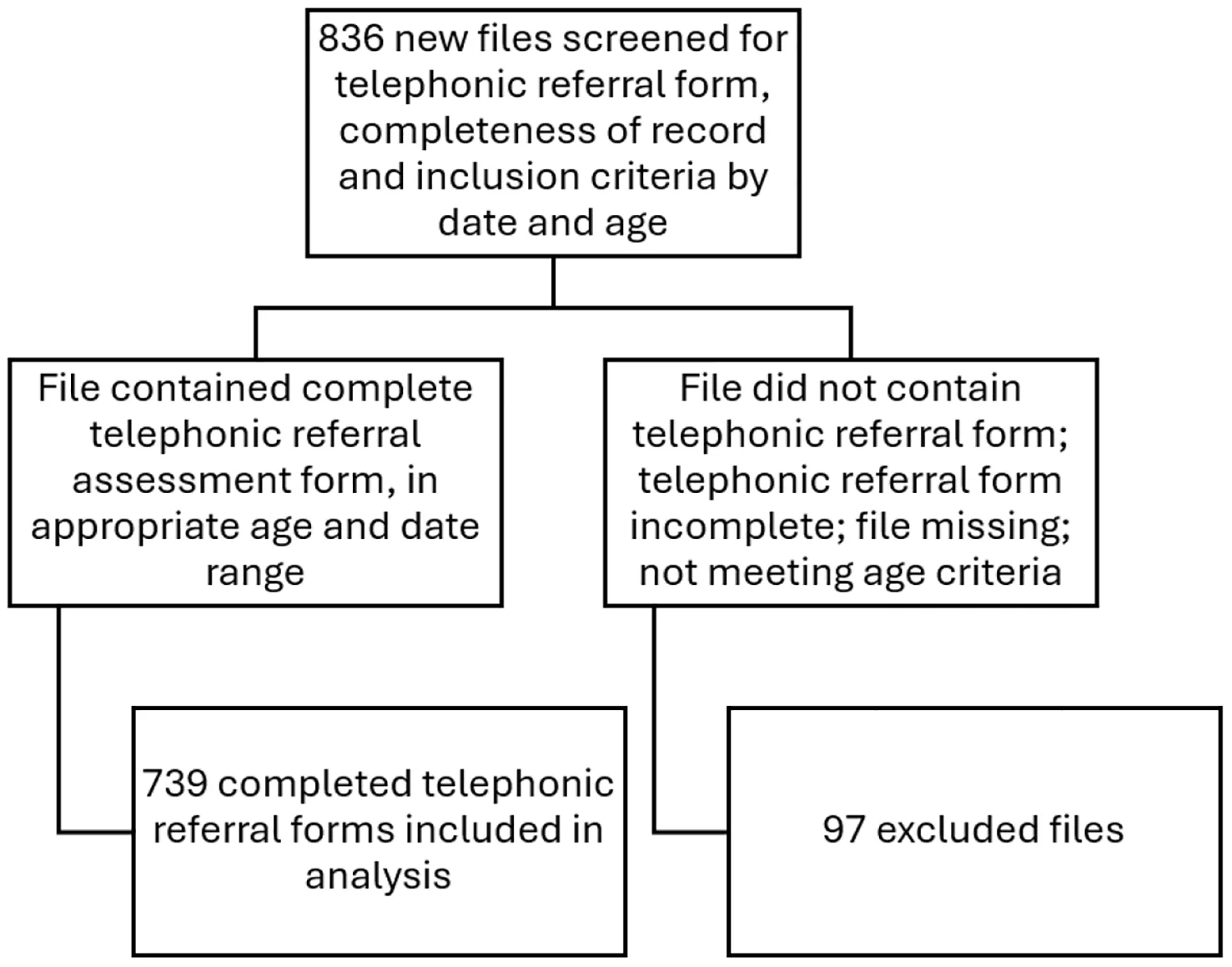
Flow chart depicting final sample of complete telephonic referral forms during the period 1 April 2019–31 March 2023.

All 836 new patient files in the Tara child and adolescent out-patient clinic, over a four-year period from beginning of April 2019 to end of March 2023 were screened for the presence of a complete paper-based telephonic referral form, which was used throughout the study period. Children under the age of 4 years old are directed to a Neurodevelopmental Pediatrician and young adults over the age of 18, and those who do not attend school are directed to their nearest community mental health clinic, therefore the age range of 4–18 years was selected for inclusion. Only complete, retrievable, telephonic intake forms for patients allocated a file number in the clinic were included. Of the 836 patients, 97 were excluded due to missing age, gender, referral source or substantially deficient symptom information. This resulted in an inclusion of a total of 739 child and adolescent telephonic referrals to the clinic in this analysis (**Figure 1**).

### Data collection and analysis

Data was collected using REDCap, directly from paper copies of the referral intake forms by eight medical students who designed the REDCap data collection tool. The REDCap template had a record identification number field (numeric number), age of the patient in years (numeric number), and gender of the patient (male or female), referral source (medical professional/parent/school/social worker), and symptoms, including neuro-developmental related symptoms, mood related symptoms, and disruptive, impulse control, and conduct symptoms. Individual symptoms, especially those relevant to child and adolescent psychiatry, were grouped broadly based on the Diagnostic and Statistical Manual (DSM 5) categorization of disorders, with depressive and bipolar related symptoms grouped as “mood disorder symptoms” (30). The reasons for referrals were also captured.

**Figure 2:**
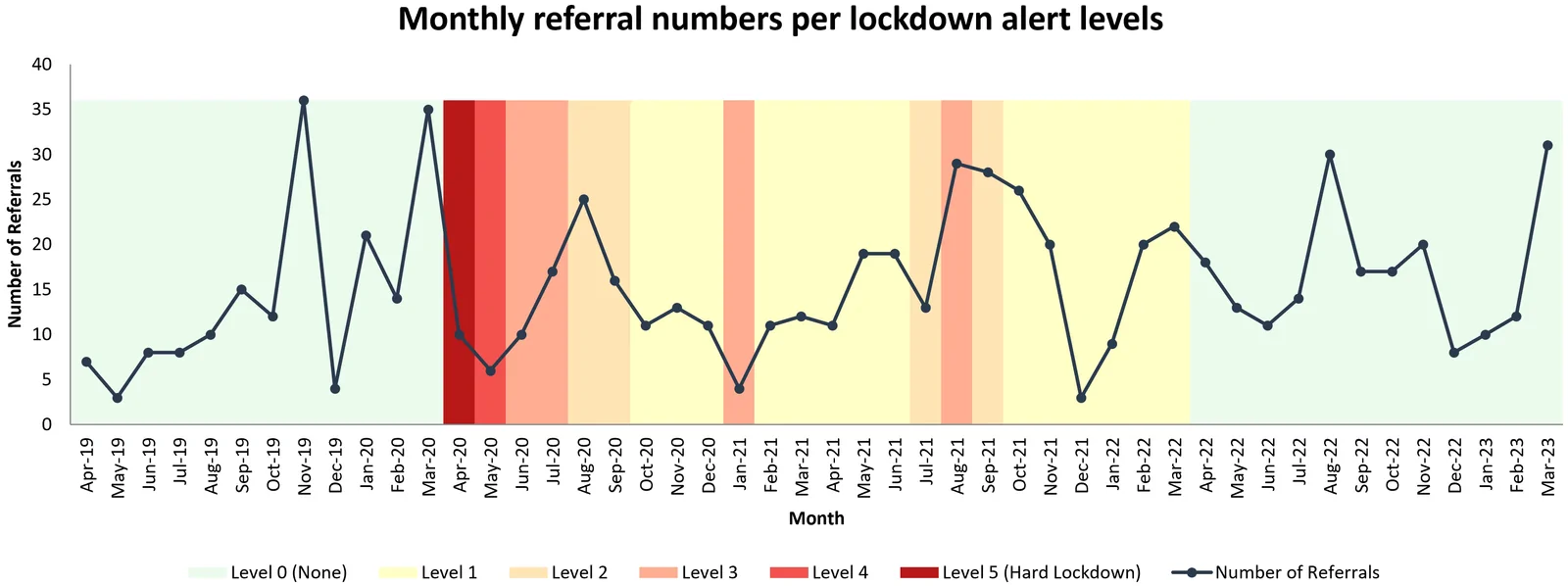
The relationship between the number of patient referrals per month and South Africa’s national COVID-19 alert levels and related lockdowns between April 2019 and March 2023.

The year and month of referral were captured, and this was used to separate data into the four-lockdown periods — pre-lockdown (April 2019 to March 2020), lockdown year 1 (April 2020-March 2021), lockdown year 2 (April 2021 to March 2022), and post-lockdown (April 2022 to March 2023). These periods corresponded to South Africa’s five-level, risk-adjusted alert level system, and associated lockdown strategy under the Disaster Management Act of 2002 where some level of lockdown was imposed from the end of March 2020 to the beginning of April 2022 (2, 3). Although the dates of lockdown and adjusted lockdown did not exactly start or end at the beginning or end of a month, the pre-lockdown, lockdown year 1, lockdown year 2, and post-lockdown time periods were simplified by a few days to the nearest month end or beginning and corresponding “COVID lockdown” year. Strict, Level 5 lockdown, alert level 5, began on 26 March 2020 and into early April 2020 and gradually eased through Levels 4 to 1 by September 2020. “Adjusted” levels were later reinstated during subsequent COVID-19 waves in 2020 and 2021, before the country returned to Level 1 in October 2021. All remaining restrictions were lifted when the national state of emergency ended on 5 April 2022, with transitional measures in place for 30 days (**Figure 2**). The stated purpose of the lockdowns was to balance level of transmission with state of health system readiness. Alert and related lockdown levels broadly corresponded to varying degrees of restricted public movement and gathering, restricted economic activity, with strict mask mandates, and social distancing requirements. Non-urgent health care was largely initially suspended and disrupted to varying degrees thereafter. Schools were closed during the highest levels of restrictions and ongoing closures persisted if social distancing requirements of each lockdown level could not be met by the school. Level 5 lockdown limited most activities, movement and interaction between people, whereas level 1 imposed minimal restrictions, where most normal activity was permitted to continue, but with ongoing mask mandates in place (2, 3, 31).

Statistical analysis was performed using SAS Version 9.4 (SAS Institute, Cary, NC, USA). The mental health referrals trend was described using a trend plot. The trend line was overlaid on the lockdown level phases to correlate the referral pattern with the lockdown levels. To examine the overall longitudinal or ordinal trajectory of mental health referrals over time, referral trend by gender, and age trend by gender, a Chi-square test for linear trend was utilized. Categorical data were summarized using counts and percentages, while continuous data were summarized using median and interquartile range (IQR) since they were non-normal. The Shapiro-Wilk test for normality was used to assess whether the numerical data were normally distributed. To compare the change in demographic distribution characteristics (age, gender, referral source, and referral reasons), between pre-lockdown, lockdown (year 1), lockdown (year 2), and post-lockdown, a Chi-square test for independence was used. To compare psychiatric symptoms across the four lockdown time points and between gender groups, a Chi-square test for independence was used. The Mann-Whitney U test was used to assess the median age differences for the most common psychiatric symptoms by gender over the four lockdown periods. A bar chart was used to show the overall gender difference in psychiatric symptoms. A significance level (α) of 0.05 was used in this study.

## Results

### Referral patterns trend analysis

The data demonstrates an initial inverse relationship between lockdown stringency and referral rates. (**Figure 2**) During periods of heightened alert levels with most severe restrictions and lockdown of movement and services, particularly alert level 5 in April and May 2020, the number of referrals decreased. As restrictions eased through alert levels 3, 2, and 1, a gradual increase in referral activity was observed. A similar pattern may have emerged in early 2021, where fluctuations in referral numbers corresponded to the reintroduction of heightened alert level 3 lockdown during a subsequent infection wave. However, this pattern is not seen with subsequent lockdown alert level adjustments later in 2021 and onwards. There was strong statistical evidence that the referrals changed in a consistent, systematic direction over time (p=0.0305).

**Figure 3:**
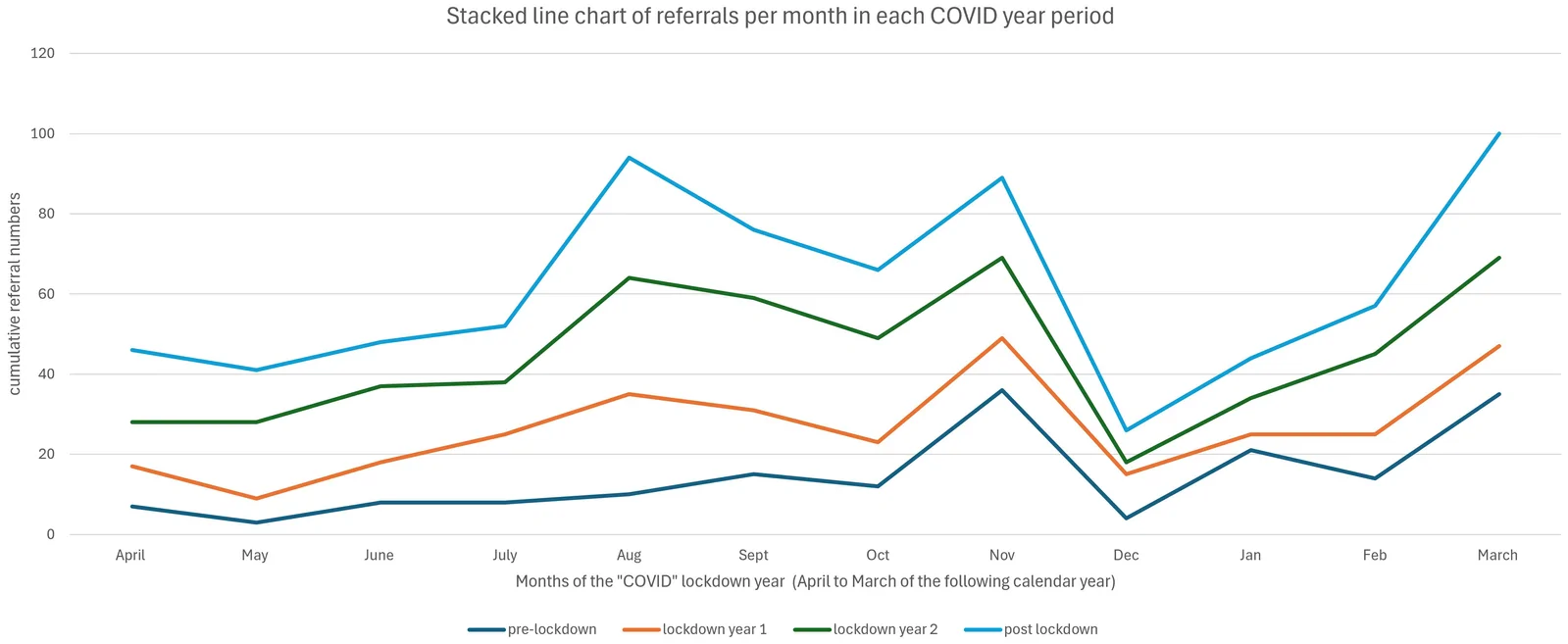
Stacked line chart depicting the cumulative number of referrals by month across the COVID year periods, Pre lockdown (April 2019-March 2020) lockdown year 1 (April 2020-March 2021) lockdown year 2 (April 2021-March 2022) post lockdown (April 2022- March 2023).

The data represented in **Figure 3** reveals substantial variability in referral activity pattern month by month over the four-year period, with some regular rise and fall patterns noted by month, with a change in pattern noted during 2020, particularly between April and June, coinciding with the initial COVID-19 level 5 alert and “hard” lockdown. (**Figures 2**, **3**) However, referrals rose consistently thereafter per month, year by year.

Of the 739 referrals over the four years, 173(23%) were referred during pre-lockdown, 146 (20%) were referred during lockdown (year 1), 219 (30%) were referred during lockdown (year 2), and 201 (27%) were referred during post-lockdown. Referral numbers declined by 15.6% lockdown (year 1) compared to the pre-lockdown level. However, referral numbers subsequently increased by 33% during lockdown (year 2) compared to lockdown (year 1). Post-lockdown referral numbers remained above pre-pandemic baseline levels. **Table 1** shows the distribution of referral proportions across demographic group characteristics.

**Table 1:** Distribution of referrals across the four study periods, with age, gender and referral source.

Demographic and referral source data	Pre-lockdown N=173	Lockdown (Year 1) N=146	Lockdown (Year 2) N=219	Post-lockdown N=201	Total
Gender					
Female, n (%)	48 (27.8)	59 (40.4)	92 (42.0)	82 (40.8)	281 (38.0)
Male, n (%)	125 (72.2)	87 (59.6)	127 (58.0)	119 (59.2)	458 (62.0)
Age in years					
Median (IQR)	9 (7-12)	12 (8-13)	10 (8-16)	10 (8-13)	10 (8-13)
Referral source					
Medical professional	21 (12.1)	37 (25.3)	44 (20.1)	57 (38.4)	159 (21.5)
Parents	2 (1.2)	7 (4.8)	2 (0.9)	1 (0.5)	12 (1.6)
School	78 (45.1)	49 (33.6)	83 (37.9)	76 (37.8)	286 (38.7)
Social worker	11 (6.4)	6 (4.1)	9 (4.1)	16 (8.0)	42 (5.7)
Other	61 (35.3)	47 (32.2)	81 (37.0)	51 (25.4)	240 (32.5)

Males constituted most referrals across all study periods overall (n = 458; 62%) compared to females (n = 281; 38%). The proportion difference between females and males was also observed across all four lockdown timepoints in this study (p-value = 0.017). However, the proportion of female referrals increased significantly from 27.8% pre-lockdown to approximately 40% during and after lockdown. There was a significant difference in referral median ages across the four study periods, (p-value=0.0016) and referral ages during lockdown year 1 were higher than in the pre-lockdown period. Regarding referrals, most of the referrals were from schools, which accounted for 38.7% overall. However, patterns were significantly different across all four time points, p-value<0.001, with the proportion of referrals from schools dropping substantially during lockdown year 1, from 45.1% to 33.6%, and the proportion of referrals from other medical professionals increasing from 12.1% to 25.3% in the same period. (**Table 1**) Most referrals throughout the study period (84%) were for assessment.

### Referral symptom analysis

Several diagnostic symptom categories demonstrated statistically significant changes across periods (**Table 2**). Overall, the most common symptoms included the neuro-developmental related symptoms (52%), mood-related symptoms (31%), and disruptive, impulse control, and conduct symptoms (31%). The neuro-developmental related symptoms dropped to 43% in lockdown (year 1) from 56% pre-lockdown and subsequently increased in lockdown (year 2), (p-value=0.042). Mood-related symptoms increased from 22% pre-lockdown to 38.4% during lockdown (year 1), (p-value = 0.005). Disruptive, impulse control, and conduct symptoms dropped from 44% pre-lockdown to 22% lockdown (year 1), (p-value<0.001). Anxiety-related symptoms changed significantly over the four years, initially decreased from 8% to 4% in lockdown (year 1) and then increased to 13% in lockdown (year 2), (p-value = 0.021). Psychosis-related symptoms rose significantly from 0.6% pre-lockdown to 6.2% during lockdown (year 1), p -value= 0.0147, although overall numbers were low. A significant change in substance related symptoms was also noted, as substance use symptoms were absent in lockdown year 1, and thereafter increased (p = 0.0008). Changes in sleep related symptoms were also significant from lockdown year 1 onwards, although overall numbers were low throughout. There were noted smaller but significant changes in other less common symptom categories, such as gender dysphoria, summarized in **Table 2**.

**Table 2:** Proportion of referrals by presenting symptom categories across the four time periods.

Symptoms	Total N(%)	Pre- lockdown		Lockdown (Year 1)		Lockdown (Year 2)		Post-lockdown		P-value
		n=173	%	n=146	%	n=219	%	n=201	%	
Neuro-developmental related symptoms	382(51.7)	96	55.5	62	42.5	110	50.2	114	56.7	**0.042**
Mood related symptoms	232(31.4)	38	22.0	56	38.4	79	36.1	59	29.4	**0.005**
Disruptive, impulse control and conduct symptoms	226(30.6)	76	43.9	32	21.9	64	29.2	54	26.9	**<0.001**
Anxiety related symptoms	71(9.6)	13	7.5	6	4.1	28	12.8	24	11.9	**0.021**
Trauma and stressor related symptoms	37(5.0)	6	3.5	13	8.9	8	3.7	10	5.0	0.095
Substance use and addiction symptoms	31(4.2)	7	4.1	0	0	16	7.3	8	4.0	**0.008**
Psychosis related symptoms	33(4.5)	1	0.6	9	6.2	14	6.4	9	4.5	**0.029**
Sleep-wake cycle symptoms	25(3.4)	0	0	9	6.2	9	4.1	7	3.5	**0.020**
Feeding and eating symptoms	18(2.4)	4	2.3	5	3.4	8	3.7	1	0.5	0.161
Elimination symptoms	14(1.9)	6	3.5	0	0	4	1.8	4	2.0	0.162
Dissociative symptoms	11(1.5)	6	3.5	5	3.4	0	0	0	0	**0.002**
Obsession and compulsion related symptoms	1(0.1)	0	0	0	0	1	0.5	0	0	0.498
Somatic symptoms	8(1.1)	2	1.2	0	0	1	0.5	5	2.5	0.106
Gender dysphoria symptoms	4(0.5)	0	0	3	2.1	1	0.5	0	0	**0.041**
*Other	43(5.8)	13	7.5	10	6.9	1	0.5	19	9.5	**0.001**
Unspecified	13(1.8)	0	0	3	2.1	7	3.2	3	1.5	0.118

*Other- includes symptoms or behavioral issues that do not fit neatly into specific diagnostic groups but were still observed among participants,representing diverse or less common psychological responses.Bold values indicate significant p values.

**Table 3**; **Figure 4** show the proportion of referrals by presenting symptom categories by gender across the four time periods. Across all four lockdown points, males had significantly higher neuro-developmental-related symptoms compared to females (61% vs 37%, p-value<0.001). Mood-related symptoms were significantly higher in females (45%) than males (23%), (p-value=0.008). Both males and females showed increased mood referrals in lockdown year 1, with further substantial increases in female mood referral numbers in lockdown year 2. All other major symptom clusters showed a decrease in number of referrals for both genders over lockdown year 1, except for trauma and stressor related symptoms. There was a rise in female presentations in lockdown year 2 across all major symptom clusters. In a trend analysis, there was an overall significant decline in the proportion of male disruptive, impulse control and conduct disorder symptom referrals from lockdown year 1 and onwards, compared to pre-pandemic numbers (p=0.0008), and a significant change in the proportion trend of anxiety symptom referrals in females over the four-year period (p=0.0253) (**Table 3**).

**Table 3:** Proportion of referrals by presenting symptom categories by gender across the four time periods.

Symptoms	Total		Pre-lockdown		Lockdown (Year 1)		Lockdown (Year 2)		Post-lockdown	
	Female n(%)	Male n(%)	Female n(%)	Male n(%)	Female n(%)	Male n(%)	Female n(%)	Male n(%)	Female n(%)	Male n(%)
Neuro-developmental related symptoms	104 (37.0)	278 (60.7)	22 (45.8)	74 (59.2)	15 (25.4)	47 (54.0)	33 (35.9)	77 (60.6)	34 (41.5)	80 (67.2)
Mood related symptoms	125 (44.5)	107 (23.4)	17 (35.4)	21 (16.8)	30 (50.9)	26 (29.9)	48 (52.2)	31 (24.4)	30 (36.6)	29 (24.4)
Disruptive, impulse control and conduct symptoms	59 (21.0)	167 (36.5)	14 (29.2)	62 (49.6)	3 (5.1)	29 (33.3)	22 (23.9)	42 (33.1)	20 (24.4)	34 (28.6)
Anxiety related symptoms	35 (12.5)	36 (7.9)	3 (6.3)	10 (8.0)	1 (1.7)	5 (5.8)	20 (21.7)	8 (6.3)	11 (13.4)	13 (10.9)
Trauma and stressor related symptoms	19 (6.8)	18 (3.9)	0	6 (4.8)	7 (11.9)	6 (6.9)	5 (5.4)	3 (2.4)	7 (8.5)	3 (2.5)
Substance use and addiction symptoms	17 (6.1)	14 (3.1)	3 (6.3)	4 (3.2)	0	0	10 (10.9)	6 (4.7)	4 (4.9)	4 (3.4)

Neuro-developmental related symptoms p-values: Total= <0.001; pre-lockdown= 0.113; lockdown year 1 = 0.001; lockdown year 2= <0.001; post-lockdown= <0.001.

Mood-related symptoms p-values: Total= <0.001; pre-lockdown= 0.008; lockdown year 1 = 0.011; lockdown year 2= <0.001; post-lockdown= 0.062.

Disruptive, impulse control and conduct symptoms p-values: Total = <0.001; pre-lockdown = 0.015; lockdown year 1= <0.001; lockdown year 2 = 0.141; post-lockdown = 0.511.

Anxiety-related symptoms p-values: Total= 0.040; pre-lockdown=0.696; lockdown year 1 = 0.226; lockdown year2 = 0.001; post-lockdown=0.593.

Trauma and stressor-related symptoms p-values: Total= 0.087; pre-lockdown=0.122; lockdown year 1 = 0.301; lockdown year 2 = 0.232; post-lockdown=0.054.

Substance use and addiction symptoms p-values: Total= 0.049; pre-lockdown= 0.362; lockdown year 2 = 0.085; post-lockdown=0.589.

**Figure 4:**
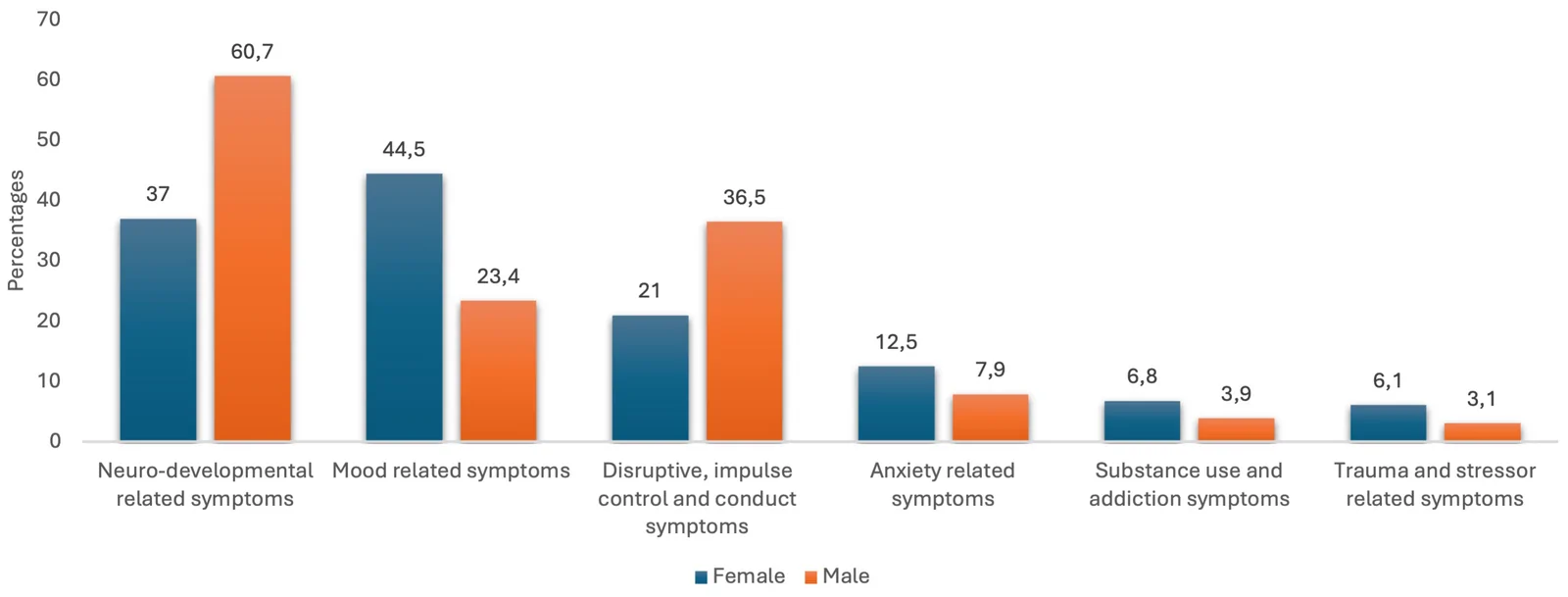
Gender differences in the number of patient referrals per major symptom category.

**Table 4** shows the referral patients’ age differences by gender for each symptom presented. Overall, there were no significant age differences for neuro-developmental related symptoms, while the median age for females with mood-related symptoms (14 years) was higher than males (12 years), (p-value<0.001). Females with disruptive, impulse control and conduct symptoms were significantly older than males, (p-value=0.03). Females with anxiety-related symptoms were also significantly older than males, (p-value=0.0012); similarly, females with trauma and stressor-related symptoms were older than males, (p-value=0.0012). The age of female referrals with mood related symptoms increased significantly in a trend analysis over the four-year period (p=0.0237) (**Table 4**).

**Table 4:** Age distribution of referrals for symptom categories by gender across the four time periods.

Symptoms	Total		Pre-lockdown		Lockdown (Year 1)		Lockdown (Year 2)		Post-lockdown	
	Median (IQR)		Median (IQR)		Median (IQR)		Median (IQR)		Median (IQR)	
	Female	Male	Female	Male	Female	Male	Female	Male	Female	Male
Neuro-developmental related symptoms	9 (7-12)	9 (7-11)	7 (6-10)	8 (7-10)	7 (6-9)	9 (7-12)	9 (7-12)	9 (7-12)	9.5 (7-12)	9 (7-11)
Mood related symptoms	14 (12-16)	12 (9-14)	12 (12-15)	10 (9-13)	12.5 (12-15)	12 (10-13)	14 (13-16)	12 (9-16)	14.5 (12-16)	12 (8-13)
Disruptive, impulse control and conduct symptoms	11 (7-13)	10 (8-12)	6 (5-12)	11 (9-12)	12 (12-16)	9 (8-11)	10 (8-12)	9 (7-12)	12 (8.5-14.5)	10 (8-13)
Anxiety related symptoms	15 (12.5-16)	11 (9-13)	6 (5-6)	10 (9-12)	16 (16-16)	11 (11-11)	15.5 (14-16)	9 (5.5-11.5)	15 (9-16)	13 (12-17)
Trauma and stressor related symptoms	13 (9-14)	10 (8-13)	-–	10 (9-12)	12 (9-14)	13 (11-14)	6 (6-11)	9 (8-13)	16 (10-16)	12 (8-13)
Substance use and addiction symptoms	14 (11-15)	16 (14-17)	17 (17-17)	15.5 (14-17)	-–	-–	11 (8-14)	15.5 (14-17)	15 (15-15.5)	16 (16-16.5)

Neuro-developmental related symptoms p-values: Total= 0.9282; pre-lockdown= 0.1663; lockdown year 1 = 0.1062; lockdown year 2 = 0.9528; post-lockdown= 0.6853.

Mood-related symptoms p-values: Total= <0.001; pre-lockdown= 0.1711; lockdown year 1 = 0.0012; lockdown year 2 = 0.1616; post-lockdown= 0.0067.

Disruptive,impulse control,and conduct symptoms p-values: Total = 0.6239; pre-lockdown = 0.1594; lockdown year 1 = 0.0277; lockdown year 2 = 0.6129; post-lockdown = 0.3581.

Anxiety-related symptoms p-values: Total= 0.0012; pre-lockdown=0.0161; lockdown year 1 = 0.0253; lockdown year2 = 0.0077; post-lockdown=0.8155.

Trauma and stressor-related symptoms p-values: Total= 0.0012; lockdown year 1 = 0.8243; lockdown year 2 = 0.4450; post-lockdown= 0.3545.

Substance use and addiction symptoms p-values: Total= 0.0833; pre-lockdown= 0.1797; lockdown year2 = 0.0061; post-lockdown=0.0398.

## Discussion

This study explored longitudinal referral patterns to a specialized child and adolescent mental health clinic in Gauteng, South Africa over the course of the COVID 19 pandemic and related lockdowns. By analyzing referrals across four defined one-year periods (pre-lockdown, lockdown year 1, lockdown year 2, and post-lockdown), the study aimed to identify changes in referral volumes, presenting symptoms, and demographic characteristics such as age and gender. The findings suggest that the longitudinal course of the COVID-19 pandemic was associated with changes in the volume and type of referrals, specifically the symptoms prompting referral, as well as the demographic patterns of referrals, with patterns largely consistent with international research (18, 20, 21, 23–25).

A variation in monthly referral patterns was noted throughout the study period, likely reflecting general patterns of child observation throughout the school academic year, with December in general experiencing fewer referrals, possibly due to closure of schools over the extended school summer holidays in the country. A temporal pattern was observed, however, between lockdown severity and referral numbers, in the period of most stringent alert levels at the beginning of the pandemic, during the period of lockdown most severely impacting on the health care system, transportation and grossly restricted personal movement, as well as rises in COVID19 hospitalizations and infections. Referrals decreased sharply during the initial alert level 5 lockdown (April-May 2020) and gradually increased as restrictions were lifted. These findings mirror international studies on referral patterns in the child and adolescent mental health setting from high income countries in the global north, showing reduced healthcare utilization during early lockdowns due to movement restrictions, fear of infection, and the reprioritization of health services (20, 21, 23, 24). Reports of disproportionally lower numbers of referrals from disadvantaged communities in a Canadian setting suggesting marginalization of already disadvantages youth, may be of heightened importance in this South African setting, although family income was not directly explored in this analysis (21). Norwegian data suggests lower healthcare utilization among youth with pre-existing mental health diagnosis during the pandemic, reflecting usage of available services and access to care patterns, rather than actual symptom pattern changes, which may be an important consideration when interpreting our data (32). There is no available data from other low- and middle-income countries.

Referral numbers later exceeded pre-pandemic levels during lockdown year 2 (29.6%) and remained elevated post-lockdown (27.2%), suggesting a lasting rise in the number of children and adolescents accessing tertiary specialized mental health care. There are few studies internationally which have reported longitudinal patterns post years 2021 and 2022, with most research focusing on the immediate changes during the early stages in the pandemic in 2020 and 2021. However, similar delayed surges in mental health presentations have been described internationally (4). A similar longitudinal analysis in Qatar over a 3-year period, which excluded the period of heighten lockdowns due to severe system disruptions, suggested significantly increased referral numbers after the pandemic lockdowns lifted (24). This sustained increase in both a high-income setting, and in the South African context suggests that the pandemic may have had an enduring impact on child and adolescent mental health patterns, due to multiple complex interacting factors with increased psychosocial upheaval, notably, disrupted schooling, social isolation, bereavement and uncertainty, and in South Africa, rising poverty and unemployment (11, 12). Covid-19 lockdown interventions had a significant impact on the financial security and employment of many caretakers, especially those working low-income jobs. Many South Africans found themselves suddenly unemployed which then may have shifted care from the private to the public healthcare sector, thereby increasing post pandemic referrals at a public health facility (33). This may have contributed to the sustained rise in referrals seen; however, this was not directly explored in our study. The proportion of referrals from medical professionals did increase in lockdown year 1 and thereafter, possibly supporting this interpretation.

Males comprised most referrals across all periods (62%), but the proportion of female referrals increased from 27.8% pre-lockdown to 40% post-lockdown. This relative rise in female presentations aligns with international trends from high income countries, consistently reporting similar gender pattern changes in referrals to child and adolescent services (18, 23, 24). There is evidence to suggest that adolescent girls experienced greater psychological distress during the pandemic, particularly anxiety and mood symptoms, partly due to disrupted social support, increased online exposure and increased risk of gender-based violence and abuse (18, 20). The rise in female referrals was however spread across the spectrum of disorders, with male youth showing a different pattern, specifically decreased referrals for externalizing symptoms. The rise in female referrals may also suggest increased help seeking behavior among females compare to males and may reflect barriers to accessing healthcare among males (25, 32).

Referral age distributions showed a statistically significant shift toward older children and adolescents during the first lockdown year. This contrasts somewhat with the Bloom et al. description of parents reporting that younger children experienced higher mental health challenges during the pandemic than older adolescents early in the pandemic and has not been reported internationally (19). Younger children may have been underrepresented during this period because school closures and limited healthcare access reduced opportunities for identification and referral. As schools reopened, referrals of younger patients increased again. The impact of school closures may also be used in the interpretation of some of the noted changes in symptom presentations in our data. During the course of the pandemic, symptom patterns overall shifted in referral intakes, with decreases in neurodevelopmental and disruptive behavioral symptoms and increases in mood and anxiety-related concerns. The reduction in neurodevelopmental, disruptive, impulse control and conduct symptoms is also in line with international referral patterns in children in adolescents from high income countries and may be linked to the pathways of referral, with school closures likely impacting on referrals from teachers (23). Our data supports this hypothesis, noting a decreased proportion of referrals from schools from year 1 lockdown, with the proportion of school referrals still not at pre-lockdown levels by the post lockdown period. Likewise, symptoms prompting referral may have been prioritized by families and caregivers based on what was impacting on day-to-day life during the pandemic or what the youth self-reported, with a lower focus on neurodevelopmental and behavioral symptoms as a cause for concerns outside the school setting (23). The rise in mood and anxiety symptoms may be less obviously linked to schools as a source of referral, however school closures may have exacerbated mood and anxiety symptoms, due to the impact of isolation, uncertainty, and disrupted routines on child and adolescent mental health as well as uncontrolled screen time and social media usage (12–15). These findings highlight the potential role of schools as early detection points for mental health difficulties and in socialization, and how their closure may have delayed access to care for young people particularly those with externalizing, attentional and behavioral problems, and impacted mental health symptoms such as anxiety and depression.

There were no substance-use-related symptom referrals during lockdown year 1, which was not sustained during subsequent years of the pandemic. This is consistent with reports nationally of decreased alcohol usage is noted in the context of restricted personal movement, and a prolonged total ban on alcohol and tobacco sales in the country during the highest alert levels (34). Small but significant changes in less common symptom presentations should be interpreted with caution. There was a significant rise in psychotic symptoms, however overall numbers of psychotic symptoms were low, as can be expected in child and adolescent groups, and so overinterpretation of this finding should be cautioned. Psychosis is multifactorial, with significant heritable and psychosocial risk factors across the life span. It is plausible that extreme psychosocial disruption may increase psychotic presentations in those at risk for psychosis (11). The direct and indirect effects of the virus on the central nervous system have also been previously implicated in neuropsychiatric presentations (7, 11, 35). There are published case series of first episode psychosis coinciding with COVID-19 infection and hospitalization (36).

Taken together, the results suggest changes in the number of referrals, the presenting symptoms, and gender and age patterns in a single tertiary specialized child and adolescent mental health state sector out-patient service longitudinally, before, during and after the COVID-19 pandemic and related lockdowns in Gauteng Province, South Africa. The patterns observed in this study closely resemble international findings and align with other South African studies that reported increased emotional difficulties during lockdown, with new evidence presented suggesting some gender and symptom patterns in this cohort (11, 15–17). These patterns of change suggest that South Africa experienced child and adolescent mental health systemic impacts during the pandemic, with possible findings relevant to the LMIC setting, in Gauteng, South Africa.

### Limitations

This study is retrospective and relies on information recorded for screening for clinical purposes and therefore subject to reliability and availability of original clinical records. The manual input of data into REDCap by multiple coders leaves room for potential transcription errors. The quality of the symptom description on the open-ended telephonic screening process is dependent on the practitioner completing the assessment form. Emergency admissions and emergency referrals are not included in this analysis, which skews the data away from the most severe, acute presentations. Taken with the missing files and incomplete referral forms, such exclusions may skew the pattern of symptoms, however data from LMIC is lacking, largely due to record keeping challenges and demand on strained health care systems, with limited health care workers per capita, therefore this analysis contributes to a better understanding of the challenges faced in this setting. The missing files and incomplete information not only complicate data validity but also reflect these ongoing system challenges and highlight areas for improvement in routine care in this setting. Finding should be interpreted in the context of these challenges.

Screening for presenting symptoms does not equate to a confirmed psychiatric diagnosis and therefore may be misleading regarding true patterns of psychiatric presentations in these children and adolescents. Data was obtained from a single specialized and public psychiatric facility, which limits the generalizability of the findings to other settings. Community child and adolescent referrals were not included in this study due to the nature of the manual referral process and health care referral system. Community based samples may be more representative of the broader community symptom patterns; however, an analysis of this nature would be difficult to reproduce outside the tertiary setting in the public health care sector in South Africa.

This study did not examine any association with actual COVID-19 infection and hospitalization in these children and adolescents and therefore cannot explore the direct and indirect biological impact of the SARS CoV2 virus on the developing child and adolescent brain and related neuropsychiatric symptoms.

### Recommendations

This review highlights some important findings over the unfolding of lockdowns surrounding the national alert levels of the COVID pandemic, guiding the direction of further enquiry specifically related to gender, age, and symptom patterns, as well as referral numbers and access to services, however significant gaps and research questions remain which require further exploration. Managing the current mental health needs of children and adolescence in the early post-pandemic period as well as planning future service delivery requires a deeper understanding of the factors contributing to any potential pattern changes which was not possible to explore with this data. Given low overall access to tertiary specialized child and adolescent services in South Africa, the question as to what the patterns of mental health related symptoms and behavior in those who were unable to access services remains prominent. Drawing from this preliminary analysis can however direct future in depth exploration of the factors contributing to gender patterns and symptom changes in referrals and access to care in this setting and assist with planning for future system responses. Policy makers should be aware of the potential gender and age-related differences and note the rise in mood and anxiety presentations. The potential protective role of schools should be highlighted in policy to protect mental health and access to care in this population.

These results emphasize the importance of considering the complexity of enduring mental health effects associated with such large-scale health care and psychosocial disruptions. Additionally, the sustained post-pandemic increase in referrals highlights the need for long-term investment in child and adolescent mental health services.

## Data Availability

The raw data supporting the conclusions of this article will be made available by the authors on reasonable request, providing local legislative, institutional and research ethics committee requirements are met.
